# Surface Electromyography of the Longissimus and Gluteus Medius Muscles in Greyhounds Walking and Trotting on Ground Flat, Up, and Downhill

**DOI:** 10.3390/ani10060968

**Published:** 2020-06-03

**Authors:** Francisco Miró, Alfonso M. Galisteo, Juan L. Garrido-Castro, Joaquín Vivo

**Affiliations:** 1 Department Comparative Anatomy and Pathology, University of Córdoba, 14071 Córdoba, Spain; an1magaa@uco.es (A.M.G.); an1viroj@uco.es (J.V.); 2 Animal Physical Therapy Service, Veterinary Teaching Hospital, Campus Universitario de Rabanales, Ctra. de Madrid Km.396, 14071 Córdoba, Spain; 3 Maimonides Biomedical Research Institute, 14004 Córdoba, Spain; juanluisgarrido@yahoo.es

**Keywords:** decline, dog, electromyography, exercise, incline, physical therapy, rehabilitation, trot, walk

## Abstract

**Simple Summary:**

In the field of canine physical rehabilitation and sports medicine, knowledge of muscle function in the therapeutic exercises prescribed is needed by physical therapists and veterinary surgeons. The longissimus dorsi and gluteus medius muscles are of great interest due to their role in locomotion related to frequent canine diseases. The muscle activity of these two muscles was studied in five Greyhound dogs performing slow, controlled leash walking and trotting on ground exercises uphill and downhill, commonly prescribed as therapeutic exercises. Results showed that for the same incline, the muscle activity of longissimus muscle was higher at the trot than at the walk. It was also shown that incline and decline affected the muscle activity of the longissimus and gluteus medius muscles of dogs walking or trotting on the ground. Walking and trotting up and downhill added separate therapeutic value to flat motion. The results of the present study might contribute to a better understanding of the function of longissimus and gluteus medius muscles in dogs, this being especially useful for the field of canine rehabilitation.

**Abstract:**

In the field of canine rehabilitation, knowledge of muscle function in the therapeutic exercises prescribed is needed by physical therapists and veterinary surgeons. To gain insight into the function of longissimus dorsi (LD) and gluteus medius (GM) muscles in dogs, five Greyhounds performing leash walking and trotting on the ground flat, up (+7%), and downhill (−7%) were studied by surface electromyography, and the mean and maximum activity was compared. For the same incline, the surface electromyography (sEMG) of LD was higher (*p* < 0.05) at the trot than at the walk. In LD muscle, trotting uphill showed significantly higher maximum activity than any other exercise. A change of +7% incline or −7% decline affected (increased or decreased, respectively) the mean sEMG of the LD and GM muscles of dogs walking or trotting on the ground. When combined, the influence of gait and incline on electromyographic activity was analyzed, and walking at certain inclines showed no difference with trotting at certain inclines. Walking and trotting up and downhill added separate therapeutic value to flat motion. The results of the present study might contribute to a better understanding of the function of LD and GM muscles in dogs, this being especially useful for the field of canine rehabilitation.

## 1. Introduction

In the past decade, knowledge of canine physical therapy has experienced growing advances, being today very helpful and established professional services complementing the multidisciplinary veterinary team. Canine rehabilitation requires multimodal treatments applied to each patient on the basis of its specific needs [1]. To be efficient, physical therapists and veterinary surgeons need a thorough and scientifically-based understanding of the kinematics and muscle function in the therapeutic exercises prescribed. Some therapeutic exercises in canine rehabilitation consist of slow, controlled leash walking and trotting flat, uphill, and downhill [2]. The lumbar and gluteal muscle regions are of great interest in canine rehabilitation due to their role in locomotion related to frequent canine diseases, such as hip dysplasia and/or osteoarthritis, spine diseases, and others. Muscles of the lumbar region are included in the epaxial systems, with the longissimus, commonly named as longissimus dorsi (LD), the more robust of them [3]. The gluteal region of the dog comprises several muscles, with the gluteus medius (GM), by far, the largest of the group [3]. The study of muscle function in quadruped locomotion has been performed by using invasive needle electromyography (EMG) and, most recently, surface non-invasive electromyography (sEMG). Some of these studies have analyzed cat locomotion [4,5,6,7,8], and the majority of them investigated horses. These studies have provided a foundation for the understanding of back and limb muscle function in quadrupeds during different exercise regimes. In horses, the EMG activity of GM muscle walking uphill [9] and the sEMG activity of lumbar muscles trotting uphill [10] have been reported. The authors in [10] highlighted the need for extra work of the hip and back extensors to increase propulsion on inclines. Other studies in horses have shown that walking and trotting downhill cause a decrease in EMG activity of the GM muscle, consistent with the reduction in propulsive forces [9]. A few studies have investigated the activity of back and/or hindlimb muscles in dogs moving on a treadmill. Some of these studies have analyzed the differences in EMG activity of back or hindlimb muscles between walk, trot, and gallop [11,12,13], finding an increase in muscle activity in the order walk-trot-gallop. By means of EMG [14,15] or sEMG [16,17,18], other studies have reported the effect of incline and decline on the activity of back or hindlimb muscles of dogs trotting and/or walking. The results of these studies have shown an increase in EMG activity in uphill movements compared with moving flat. Some of these studies have shown a decrease in muscle activity walking downhill compared with walking flat, but one of them [16] has not found differences between walking downhill and walking flat. Other studies have reported the sEMG activity of some hindlimb muscles of dogs with hip osteoarthritis walking on a treadmill [19] or evaluated the changes in back muscle activity related to hindlimb lameness [20]. Recently, a systematic review of sEMG in animal biomechanics has been published [21]. A comparison of on-ground and treadmill kinetic [22] and kinematic [23] of the canine hindlimb has shown similarities but also significant differences.

Although investigations have suggested that walking and trotting uphill could strengthen the gluteal and lumbar muscles in dogs, this idea is not yet sufficiently explored. Furthermore, we do not have sufficient knowledge of the activity of LD and GM muscles of dogs walking and trotting downhill. To the authors’ knowledge, there is little scientific evidence regarding the muscle activity of the LD and GM muscles of dogs during on-ground walking and trotting. 

We hypothesized that sEMG activities of LD and GM muscles would be higher during trotting than during walking and that sEMG activity of LD and GM muscles would increase when the dogs were uphill and decreased when they were downhill. The purpose of the present study was to gain further insight into the muscle activity of LD and GM muscles of dogs walking and trotting on the ground flat, uphill, and downhill. 

## 2. Materials and Methods 

### 2.1. Dogs

Five healthy adult Spanish Greyhounds, similar in size and shape (mean body weight 27.44 ± 1.04, height at the withers 65.10 ± 0.96, and age 3.60 ± 0.65 years), were included in the study. A complete orthopedic and physical examination confirmed that they were free of any pain or lameness. All dogs had similar house conditions and daily exercise. This study did not include an invasive method; it was performed according to the ethical guidelines for animal experimentation, and it was approved by the Committee on Bioethics for Animal Experimentation of Córdoba University, Spain (reference number, 3811). Five days a week, during the 4 weeks prior to data acquisition, the dogs were accustomed to walk and trot on a leash at a comfortable speed on the recording area by an experienced handler. The recording area comprised horizontal and incline (7%) concrete walkways.

### 2.2. Data Collection 

**Figure 1:**
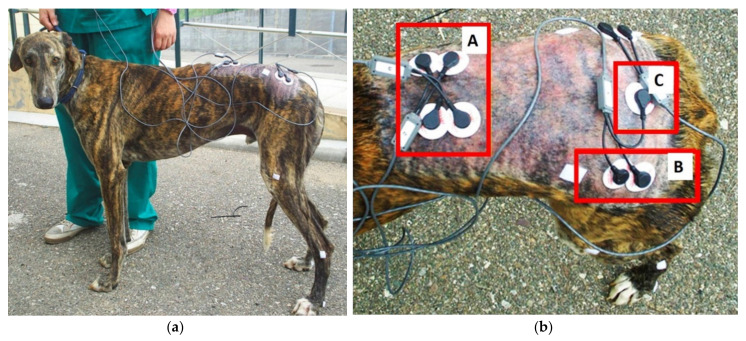
Lateral (**a**) and dorsal views (**b**) to illustrate the placement of surface electrodes. A, electrodes over longissimus dorsi muscles. B, electrodes over left gluteus medius muscle. C, reference electrode over spinous processes of the sacrum.

The sEMG activity of all the dogs was collected during the same session. The skin of the gluteal and lumbar regions on both left and right sides was suitably prepared for optimal electrode adhesion by shaving, cleaning with soapy water and alcohol, and drying. Surface adhesive electrodes (Ag/AgCl, 4 mm diameter, Lessa^®^, Lessa-AB group S.A., Barberà del Vallès, Spain) were fixed in bipolar configuration over the gluteal, lumbar, and sacral regions (Figure 1). A pair of electrodes was glued parallel to the muscle fiber direction of LD, 3 cm laterally to the 4th to 6th spinous processes of lumbar vertebrae. Another pair of electrodes was glued, parallel to the muscle fiber direction of the GM, midway between the iliac crest of the hip bone and the greater trochanter of the femur. A single reference electrode was placed over the spinous processes of the sacrum. To determine the time events, circular markers were glued on to the skin over the base of the fifth and second metatarsal bones of both hindlimbs. Before the exercises, the sEMG activities of the LD and GM muscles were recorded for several minutes with dogs in standing position. After gentle warming up at walk and trot, the sEMG activities of left and right LD and GM muscles of every dog were simultaneously recorded at different exercises. The dogs were led on a leash by the handler at the walk and trot at their comfortable speed on the ground flat, uphill (7%), and downhill (7%). All exercises were performed in the same order, with a 3 min break between exercises. Several trials at each exercise were recorded.

The sEMG measurements were obtained by an Electromyography telemetric system (Telemyo^®^ 2400 T, Noraxon Inc. 13430 N. Scottsdale Rd, Suite 104, AZ 85254, USA) at a sampling frequency of 1500 Hz. Wires containing a preamplifier (×500) and a filter (high-pass 10 Hz, low-pass 500 Hz) connected the electrodes to the transmitter, tied to the handler’s waist. Raw sEMG data were transmitted by telemetry to a computer (TOSHIBA^®,^ Satellite A45 series, Toshiba Electronics Europe GmbH España, San Fernando de Henares, Spain), where the specific software (Noraxon Mioresearch XP, Noraxon Inc. 13430 N. Scottsdale Rd, Suite 104, AZ 85254, USA) received, processed, and analyzed them. A video camera (Sony^®^, handycam DCR-HC23, Sony Europe B.V. España, Barcelona, Spain), perpendicular to the central area of the dog’s line of motion, synchronized by the software, enabled to relate the sEMG data to the time events of the corresponding hind limb. The body of the camera was 1 m height, and the zoom lens was adjusted to allow a 4-m field of view. The shutter speed was 50 fps, and the frame rate was used to calculate temporal measurements.

### 2.3. Data Processing and Analysis

By means of the previously mentioned software, raw sEMG signals (in microvolts, µV) were rectified, smoothed (RMS-100ms), and filtered (low pass, Butterworth, cut-off frequency 50 Hz). Video images of the trials were studied, and the best two to three consecutive strides per trial were selected. Strides selected from trials were those in which dogs moved steadily and straight, at a comfortable speed, over the central area of the walkway. Then, the sEMG activity of ten selected strides from right and left hindlimbs per exercise was analyzed. The initial hindlimb’s paw contact and heel liftoff, detected visually—helped by the position of the marker over the osseous references, were the beginning of the stride and the end of the stance phase, respectively. The previous frame to the initial ground contact was the end of the stride.

Speed, stride duration, and stance phase duration (in the percentage of the stride duration) were measured from the video images, based on film-frame events. The mean and maximum sEMG activity of ten consecutive seconds of the central and stable area of muscle activity, while the dogs were standing, was analyzed. The maximum and mean sEMG activities for every exercise were obtained. Mean and standard deviation (SD) of these variables were obtained and expressed in tables and graphically represented in bar charts. The sEMG data were analyzed using mixed model analysis, with the mean and maximum sEMG activities as the response variables, exercise and muscle as the fixed effects, and subject as the random effect. Post hoc multiple comparisons between exercises were conducted with a Tukey’s test. Statistical significance was identified at a level of 0.05. Data were processed and analyzed by using IBM-SPSS^®^, the 25th version (Armonk, NY, USA).

## 3. Results

Results (mean ± standard deviation-SD) for speed, stride duration, and stance phase duration measured in the present study are shown in Table 1. 

The speed of movement decreased when dogs moved uphill and downhill with respect to flat movements at their respective gait. The stride duration and the stance phase duration were significantly higher in all the walks than in all the trots. The values of stride duration were significantly higher in the dogs walking uphill compared with those walking downhill. This variable was not affected by the incline or decline of the ground in the trots. The stance phase duration walking uphill was significantly higher than walking downhill.

**Figure 2:**
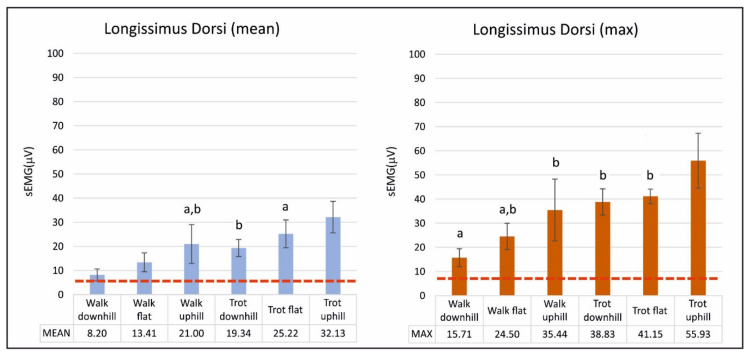
Graphical representation (mean ± SD) of mean and maximum surface electromyographic activity of longissimus dorsi. Analysis of five Spanish Greyhounds standing, walking, and trotting on the ground flat, up (+7%), and downhill (−7%). Horizontal, dashed line represents a mean sEMG activity of dogs standing. Bars sharing the same letter are not significantly different according to Tukey’s post hoc test (*p* < 0.05).

The results of mean and maximum sEMG activities (mean ± SD) of the LD muscle are reported numerically in Table 2 and graphically in the bar chart of Figure 2.

For the same gait, the mean sEMG activity increased significantly when dogs were uphill and decreased when they were downhill. For the same gait, in maximum values, significant differences were found only between trotting flat and uphill. The walks, except the walk uphill, had significantly lower mean sEMG activity than the trots. For the same incline, the mean sEMG activity was always significantly higher at the trot than at the walk. The same was found in maximum activity; however, differences between walking flat and trotting flat were not significant. Trotting uphill showed significantly higher maximum activity than any other exercise.

**Figure 3:**
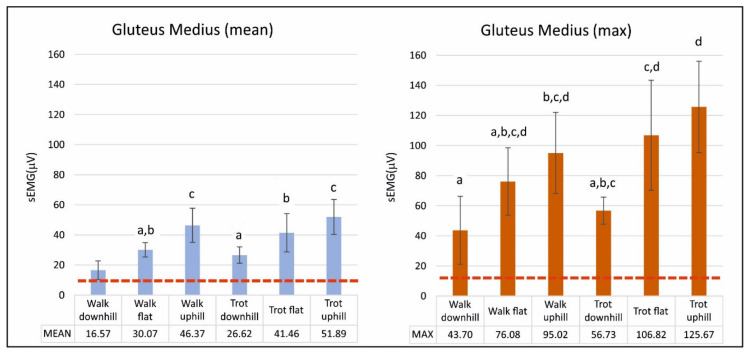
Graphical representation (mean ± std) of mean and maximum surface electromyographic activity of gluteus medius muscle. Analysis of five Spanish Greyhounds standing, walking, and trotting on the ground flat, up (+7%), and downhill (−7%). Horizontal, dashed line represents a mean sEMG activity of dogs standing. Bars sharing the same letter are not significantly different according to Tukey’s post hoc test (*p* < 0.05).

The results of mean and maximum sEMG activities (mean ± SD) of the GM muscle are reported numerically in Table 3 and graphically in the bar chart of Figure 3.

Within the same gait, the mean sEMG activity increased significantly when the dogs were uphill and decreased when they were downhill. The same was found in maximum activity; however, differences were not significant. For the same incline, the mean sEMG activity was not significantly higher at the trot than at the walk, except in downhill. 

## 4. Discussion

The present study analyzed muscle activity in slow, controlled leash walking and trotting exercises, commonly prescribed as therapeutic exercises in dogs undergoing physical rehabilitation. Kinematic [23] and kinetic [22] similarities but also relevant differences have been found in the gait pattern of the hind limb of dogs walking on the ground and on the treadmill. According to the purpose of the present study, the sEMG activity was assessed on ground exercises. Our results confirmed that gait, incline, and decline influenced the sEMG activity of LD and GM muscles of the dogs walking and trotting on the ground.

In contrast to the invasive needle or fine wire EMG, where the probes can be placed exactly into specific parts of the muscles, non-invasive sEMG signals represent a sum of the target and nearby muscle activities [24]. In the present study, the location of the electrodes was based on previously reported studies [16,17,18,19], and the sEMG signals elicited were supposed to come mostly from the LD and GM. Before the study, several tests were performed to analyze the characteristics of the sEMG signal. To a sampling frequency of 1500 Hz, a low pass Butterworth filter, with a cut-off frequency of 50 Hz was chosen to reduce the high-frequency noises while retaining the true information of the EMG signal. The sEMG has proven to be a valid method to give useful and valuable information of locomotion in equines [10,25,26] and dogs [16,19,24,27,28]. Determining the timing of excitation and magnitude of muscle activation can provide an accurate picture of the function of muscles during movement [27]. Timing of excitation, when muscles turn “on” or “off” during the gait cycle, was out of the scope of the present article. To provide a measure of the magnitude of muscle force, the mean and maximum sEMG activities were calculated. The maximum and mean muscle activities may be useful for the therapist to establish balanced rehabilitation programs focused on gaining muscle strength and endurance. In order to avoid factors related to sex, weight, height, and morphometry, dogs included in the study were all male of the same breed, similar age, weight, and conformation.

The speed of movement decreased when dogs moved uphill and downhill with respect to flat movements at their respective gait. These results were in concordance with those previously reported for walking cats [5] and dogs [2]. In quadrupedal mammals, the stance phase duration of the hind limbs has been commonly associated with propulsive force generation [24,25,27]. An increase in stance phase duration obtained in the present study in uphill movements might be consistent with the need to increase time for propulsion. However, neither walking nor trotting downhill −7% changed the percent value of time devoted to the stance phase. These results were in concordance with those reported for hounds walking downhill −5% on a treadmill [25]. In contrast, other authors have shown that cats walking downhill reduce the percent of stance [6]. In the study cited above, the grade of decline was steeper (25% to 100%) than in the present study (−7%). In addition to possible interspecific differences, we supposed that the degree of decline might also affect this parameter differently.

### 4.1. Longissimus Dorsi Muscle

Epaxial muscles, dorsal to the vertebral column and ribs, are the only muscles with the anatomical configuration necessary for the extension of the back [29]. Epaxial muscles of mammals have been suggested to mobilize and globally stabilize the trunk, but timing and degree to which they serve a particular function likely depend on the gait performed and the vertebral level [12]. At the lumbar level, epaxial muscles resist sagittal flexion more and possess a greater extensor role than in other axial parts [12]. In lame dogs, the increased need to stabilize the trunk changes the sEMG patterns of the ipsilateral and contralateral LD muscles [20]. The level of recruitment of epaxial muscles had been found to be significantly higher during trotting than during walking [12]. In the present study, for the same incline, the mean sEMG activity was always significantly higher at the trot than at the walk. This was probably due first to the increased need for stability on the sagittal plane of the axis and second to the increased need for the extensor function for propulsion. Based on the results of the present study, transition from walk exercises to trot exercises at the same incline could be considered an effective form of increasing workload to strengthen the lumbar muscles. 

As shown in cats [7], the present study showed the mean sEMG activity of LD muscles of the dogs significantly higher during walking uphill than during walking flat. The mean sEMG activity walking downhill was significantly lower compared with walking flat. Similarly, the results of mean sEMG activity in the trot showed an increase in the uphill and a decrease in the downhill. Besides, the maximum sEMG activity was significantly higher when trotting uphill than in any other exercise. These findings were coincident with what has been found in horses [10] and dogs [14] trotting on treadmills. As recommended by some authors [30], walking and trotting uphill may be useful to strengthen the lumbar muscles for those dogs suffering from spine diseases. In our opinion, the progression from moving flat to uphill at slight inclines at the same gait would be the logical progression in the early phases of rehabilitation. Proprioceptive feedback from the limbs and trunk is one of the systems that the nervous system integrates to control locomotion. Proprioception allows an animal to stabilize itself against perturbations, such as caused by inclines. It has been suggested that the nervous system uses different motor programs to successfully control locomotion on the level, incline, and decline [2,5,6,7]. While walking or trotting uphill may be considered a concentric isotonic exercise, moving downhill is an isotonic but eccentric exercise [16] with a more complex neural control [5,6,8]. The automated movement displayed on flat moving becomes a more intentional motion during walking downhill [2]. Many therapeutic exercises prescribed for geriatric patient focus not only on strengthening but also, and of crucial importance, on enhancing coordination and proprioception [31,32,33]. Whenever possible, walking and trotting uphill and downhill at a slight incline should be recommended to dogs suffering from proprioceptive deficits. When combined, the comparison of gait and incline was analyzed in the present report, and walking uphill did not show significant differences, with trotting flat and trotting downhill, in mean and maximum sEMG activities. In canine rehabilitation, choosing between one of these exercises would depend on the stage of rehabilitation and patient’s conditions [1].

### 4.2. Gluteus Medius Muscle

GM muscle is anatomically positioned to produce limb retraction [11], acting primarily on the hip joint by means of inserting on the greater trochanter of the femur [3,13]. Gluteus medius, together with the hamstring muscles, actively stabilize the hip joint at the end of the swing phase and in the early stance of the ipsilateral hindlimb cycle [11,13]; afterward, during the following period of stance, the hip joint is extended to generate propulsive forces to pull the trunk forward [6,9,13,25,27]. 

In dogs, stabilizing and propulsive functions of GM muscle are present in the walk, trot, and gallop, but the level of excitation increases in the order walk-trot-gallop due to greater propulsive impulses that are required [11]. Our results did not show that for the same incline, mean and maximum were significantly higher at the trot than at the walk, except for the mean sEMG downhill.

In the present study, the mean sEMG activity of GM muscle of the dogs walking uphill increased significantly compared with the dogs walking flat. These findings were in agreement with those reported for cats [4,5,7], hounds [16], and horses [9]. The latter authors have stated that hip retractors are the primary muscles responsible for powering locomotion in response to increasing workload. In our opinion, the uphill-related changes found in the mean sEMG activity of GM muscle at the walk were consistent with the need for increased propulsion. As it has been found in dogs during walking exercises [18], in the present study, the most remarkable difference was found when comparing uphill and downhill. A common finding on physical examination when evaluating dogs suffering from hip diseases is atrophy of gluteal muscles. In view of the above, we agree with some authors [30,34] in recommending walking uphill to strengthen the gluteal muscles of dogs suffering from this disease. According to studies of joint kinematics, walking uphill has also been recommended as a means of strengthening muscles that cause flexion of the hip joint [2]. For walking uphill, the hind paws need to be elevated to greater heights to clear the inclined walkway [5], and an increase in the need for flexion of the hip joint is required [2]. 

It has already been reported in cats [8] and horses [9] that walking downhill causes a decrease in the sEMG activity of GM muscle. Moving downhill causes the body mass to shift in a forward direction, and less propulsive power is required by the hind limbs. The downhill-related changes found in the activity of GM muscle of dogs, in the present study, at the walk might be related to a decrease in the propulsive activity. In contrast, other authors have reported that dogs walking downhill –5% do not represent a change in the level of activity of GM muscle [16]. They have indicated that a mild degree of negative inclination and slow speed of the treadmill might have been insufficient to modify the activity of the GM muscle. In our opinion, differences between experimental protocols, treadmill exercises vs. on ground exercises, could possibly explain differences of the results. In older patients’ muscle mass, coordination and proprioception decrease with age [33]. In them, disorders, such as hip osteoarthritis, even subclinical, lead to changes in the locomotor pattern [19] caused by changes in the neural control of locomotion, being logical to dedicate a portion of the exercise program to focus on proprioception and balance [34]. It has been studied that downhill movement provokes changes in kinematics that alter the neural control of motion of limbs and trunk [6,8]. In our opinion, walking downhill at a slight decline would improve the balance and proprioception of geriatric patients and those with hip diseases. Combined with walking uphill, it would improve hip joint range of motion and would strengthen flexor and extensor muscles of the hip, to complete motor reeducation. Data has been published about the influence of inclination on limb joint kinematics in healthy [2] and osteoarthritic [28] dogs walking on the ground, uphill, and downhill. Each exercise has specific effects on joint kinematics, which must be considered when planning a rehabilitation program [28,34], to prevent possible damage to hip and other joints.

Similar to what has been previously reported for horses [26], cats [4], and dogs [15], in the present study, the mean sEMG activity of the GM muscle of dogs trotting uphill increased. In agreement with earlier results in dogs [15] and horses [9], trotting downhill caused a significant decrease in the mean activity of the GM muscle of dogs in the present study. As it has been mentioned earlier for the walk, trot uphill would cause an increase of propulsive force in hindlimbs to act against the backward component of gravity, and trot downhill would decrease propulsive force, but it would demand greater neural control. For those dogs suffering from hip diseases, trotting uphill is useful to strengthen the gluteal muscles [30]. Furthermore, trotting downhill would improve balance and proprioception for arthritic and/or geriatric patients. However, from our point of view, trotting uphill or downhill should be recommended once locomotion has improved sufficiently during trotting flat, which, in terms of some authors, is to maintain the correct gait pattern throughout the exercise [31]. When combined, the comparison of gait and incline was obtained in the present report, and there were no statistical differences between the mean sEMG activity of GM muscle of dogs walking flat and trotting downhill and flat and between walking uphill and trotting uphill. Although with no significant differences in some cases, the maximum sEMG activity was higher in dogs trotting uphill. In canine rehabilitation, kinematics of limb and trunk during exercises and each patient’s need should be considered to select the right exercise regimens. 

From this study, physical therapists and veterinary surgeons might improve their knowledge of the muscle function of dogs walking and trotting on the ground flat, uphill, and downhill. Only five dogs of the same breed were analyzed in the present study. Therefore, the results should be interpreted with caution. It is likely that dogs of other conformation would have different muscle activity. Investigations on a larger sample and on other breeds would provide a more comprehensive understanding of the muscle activity of the LD and GM muscles of dogs walking and trotting on the ground flat, up, and downhill.

## 5. Conclusions

The present study confirmed first that for the same incline, the mean sEMG activity of LD of dogs was higher at the trot than at the walk. At the same incline, the transition from walk exercises to trot exercises could be considered an effective form of increasing workload to strengthen the lumbar muscles. Second, the mean sEMG activity of LD and GM muscles was significantly higher in dogs walking and trotting uphill than in their respective flat gait. For dogs suffering from spine and hip diseases, change from flat to uphill at slight inclines in the same gait would be the logical progression in early phases of rehabilitation. Third, walking and trotting uphill and downhill at a slight incline should be recommended to dogs suffering from proprioceptive deficits. Choosing between some of these exercises would depend on the stage of rehabilitation and the patient’s conditions.

## Figures and Tables

**Table 1:** Results (mean ± SD) of speed, stride duration, and stance phase duration of five Spanish Greyhounds walking and trotting on the ground flat, up (+7%), and downhill (−7%).

Exercise	Speed (m/s)	Stride Duration (s)	Stance Phase Duration (%)
Walk downhill	1.00 ± 0.13	0.80 ± 0.11 ^a^	63.07 ± 2.01 ^a^
Walk flat	1.40 ± 0.15	0.78 ± 0.06 ^a^^,b^	62.25 ± 1.45 ^a^
Walk uphill	0.94 ± 0.11	0.82 ± 0.10 ^b^	64.66 ± 2.54
Trot downhill	2.07 ± 0.32	0.51 ± 0 ^c^	44.23 ± 3.08 ^b,c^
Trot flat	2.93 ± 0.29	0.49 ± 0.02 ^c^	42.70 ± 3.70 ^b^
Trot uphill	1.84 ± 0.30	0.52 ± 0.03 ^c^	43.08 ± 4.39 ^c^

In columns, the values sharing the same letter are not significantly different, according to Tukey’s post hoc test (*p* < 0.05).

**Table 2:** Mean and maximum surface electromyographic activities (mean ± SD, µv) of longissimus dorsi of five Spanish Greyhounds walking and trotting on the ground flat, up (+7%), and downhill (−7%).

Exercise	Mean	Maximum
Standing	6.56 ± 1.1	
Walk downhill	8.20 ± 2.45	15.71 ± 3.73 ^a^
Walk flat	13.41 ± 3.88	24.50 ± 5.45 ^a,b^
Walk uphill	21.00 ± 8.02 ^a,b^	35.44 ± 12.82 ^b^
Trot downhill	19.34 ± 3.55 ^b^	38.83 ± 5.40 ^b^
Trot flat	25.22 ± 5.77 ^a^	41.15 ± 2.99 ^b^
Trot uphill	32.13 ± 6.54	55.93 ± 11.40

The values sharing the same letter are not significantly different, according to Tukey’s post hoc test (*p* < 0.05).

**Table 3:** Mean and maximum surface electromyographic activity (mean ± SD, µv) of gluteus medius muscle of five Spanish Greyhounds walking and trotting on the ground flat, up (+7%), and downhill (−7%).

Exercise	Mean	Maximum
Standing	10.82 ± 4.76	
Walk downhill	8.20 ± 2.45	43.70 ± 22.54 ^a^
Walk flat	16.57 ± 6.17 ^a, b^	76.08 ± 22.40 ^a, b, c, d^
Walk uphill	46.37 ± 11.32 ^c^	95.02 ± 27.00 ^b, c, d^
Trot downhill	26.62 ± 5.46 ^a^	56.73 ± 9.02 ^a, b, c^
Trot flat	41.46 ± 12.80 ^b^	106.82 ± 36.60 ^c, d^
Trot uphill	51.89 ± 11.65 ^c^	125.67 ± 30.33 ^d^

The values sharing the same letter are not significantly different, according to Tukey’s post hoc test (*p* < 0.05).
